# Distributional analysis of semantic interference in picture naming

**DOI:** 10.1080/17470218.2016.1165264

**Published:** 2017-04-01

**Authors:** Ardi Roelofs, Vitória Piai

**Affiliations:** a Donders Centre for Cognition (DCC), Radboud University, Nijmegen, The Netherlands; b Department of Psychology and Helen Wills Neuroscience Institute, University of California, Berkeley, CA, USA

**Keywords:** Naming, Picture–word interference, Response time distribution, Semantic interference

## Abstract

In picture–word interference experiments, participants name pictures (e.g., of a cat) while trying to ignore distractor words. Mean response time (RT) is typically longer with semantically related distractor words (e.g., *dog*) than with unrelated words (e.g., *shoe*), called semantic interference. Previous research has examined the RT distributional characteristics of distractor effects by performing ex-Gaussian analyses, which reveal whether effects are present in the normal part of the distribution (the μ parameter), its long right tail (the τ parameter), or both. One previous study linked the semantic interference effect selectively to the distribution tail. In the present study, we replicated the semantic interference effect in the mean picture naming RTs. Distributional analysis of the RTs and those of a previous study revealed that semantic interference was present in both μ and τ. These results provide evidence that the effect is not selectively linked to the τ parameter, and they warn against any simple one-to-one mapping between semantic interference and distributional parameters.

An important tool in studying spoken word production is the picture–word interference paradigm, which has been used to obtain evidence from healthy adult speakers (e.g., Damian & Martin, 1999; Schriefers, Meyer, & Levelt, 1990) and from impaired populations, including people with aphasia as a consequence of stroke (e.g., Hashimoto & Thompson, 2010) or neurodegenerative disease (e.g., Thompson et al., 2012). In this paradigm, speakers name pictures while trying to ignore spoken or written distractor words. For example, they say “cat” to a picture of a cat, while trying to ignore the superimposed written word *dog* (the semantic condition), the word *shoe* (the unrelated condition), the word *cat* (the identity condition), or a row of Xs (the neutral condition). Previous research (e.g., Damian & Martin, 1999; W. R. Glaser & Düngelhoff, 1984; W. R. Glaser & Glaser, 1989; Rayner & Springer, 1986; Roelofs, 2007; Starreveld & La Heij, 1996) has shown that mean response time (RT) is longer on semantic than on unrelated trials, called *semantic interference*. Moreover, RTs are longer on unrelated than on neutral trials, and they are shortest on identity trials. Picture–word interference effects are related to colour–word Stroop effects (e.g., W. R. Glaser & Glaser, 1989; Roelofs, 2003). In the Stroop task, individuals name the presentation colour of printed incongruent or congruent colour words (e.g., the words *red* or *green* in green colour; say “green”) or neutral Xs. Alternatively, participants name colour patches with superimposed incongruent words, congruent words, or Xs (e.g., M. O. Glaser & Glaser, 1982). Mean RT is typically longer on incongruent than on neutral trials, which is called *Stroop interference*. Moreover, mean RT is often shorter on congruent than on neutral trials, which is descriptively called *Stroop facilitation* (see MacLeod, 1991, for a review). In picture–word interference, corresponding effects are obtained—namely, longer RTs on semantically related trials than on neutral trials, henceforth *Stroop-like interference*, and often shorter RTs on identity trials than on neutral trials, henceforth *Stroop-like facilitation* (e.g., W. R. Glaser & Düngelhoff, 1984; Roelofs, 2003).

Whereas researchers have relied heavily on mean RT in studies of picture–word interference and colour–word Stroop task performance (e.g., MacLeod, 1991), some previous studies have performed ex-Gaussian analyses to characterize entire RT distributions. RTs are typically not normally distributed but their distributions are positively skewed (i.e., the distribution tail is longer for the slow responses than for the fast responses). The ex-Gaussian function consists of a convolution of a Gaussian (normal) and an exponential distribution, which generally provides good fits to empirical RT distributions (e.g., Luce, 1986; Ratcliff, 1979). The function captures both the normal part and the longer right tail of a distribution. An ex-Gaussian analysis provides three parameters—namely, μ and *σ* reflecting the mean and standard deviation of the Gaussian portion, and τ reflecting the mean and standard deviation of the exponential portion. Theoretically, the mean RT equals the sum of μ and τ, so that ex-Gaussian analyses decompose the mean into two additive components, which characterize the leading edge (μ) and the tail (τ) of the underlying RT distribution. Effects in μ indicate that an experimental manipulation leads to a shift of the entire RT distribution of one condition relative to another, whereas effects in τ indicate that a manipulation leads to distributional skewing (see Balota, Yap, Cortese, & Watson, 2008, for an extensive discussion). In the early days of experimental psychology, Wundt ran an extensive research programme examining psychological processes by means of RT measurements, with his student Cattell making several seminal observations on the time it takes to name pictures and colours (e.g., Cattell, 1886). Wundt argued that deviations from normality of RT distributions reflect fluctuations or lapses of attention (Wundt, 1918).

In a seminal study of the Stroop task using ex-Gaussian analysis, Heathcote, Popiel, and Mewhort (1991) observed that the RT effect of distractor condition (i.e., incongruent, congruent, neutral) may be different for the condition means and the three ex-Gaussian parameters. Mean RTs were longer for incongruent trials than for congruent and neutral trials, while congruent and neutral trials did not differ from each other. However, μ was larger for incongruent than for neutral trials and smaller for congruent than for neutral trials. Moreover, τ was larger for congruent and incongruent trials than for neutral trials, whereas congruent and incongruent trials did not differ. Thus, relative to neutral trials, incongruent trials showed interference in both μ and τ, whereas congruent trials showed facilitation in μ and interference in τ. Because the interference and facilitation had about the same magnitude, no difference between congruent and neutral trials was obtained in the mean RTs. These results have been replicated by Mewhort, Braun, and Heathcote (1992), Spieler, Balota, and Faust (1996, 2000), and Roelofs (2012).

Ex-Gaussian analyses have also been performed on the RTs of some picture–word interference studies to examine the distributional characteristics of semantic interference in picture naming (for distributional analyses of semantic facilitation in picture and word categorizing, see Roelofs, 2008). In a picture–word interference study with semantically related and unrelated distractor words, Piai, Roelofs, and Schriefers (2011, Experiment 1) obtained semantic interference in the mean RTs, which was reflected in the μ but not in the τ parameter. Moreover, in another study, Piai, Roelofs, and Schriefers (2012, Experiment 2) observed that semantic interference in mean RTs was reflected in μ or τ depending on the visibility of the distractor words. These results indicate that semantic interference may be evident in μ as well as in τ.

More recently, Scaltritti, Navarrete, and Peressotti (2015) conducted picture–word interference experiments that included semantic, unrelated, neutral, and identity conditions (they also examined the effect of distractor frequency, which is not relevant for now). In one experiment (Experiment 1), semantic interference was present in τ and only marginally in μ, whereas Stroop-like facilitation (identity vs. neutral) was present in μ as well as in τ. In another experiment without identity distractors (Experiment 3), semantic interference was present in τ but not in μ. The stimuli used to assess the semantic effects in the two experiments were the same, but different groups of participants were tested. Based on their results, Scaltritti et al. argued that semantic interference is specifically linked to the τ parameter: “semantic interference is mainly mediated by the exponential component of the RT distribution” (p. 1355). They stated, “the semantic interference effect seems to selectively involve the slowest RTs and only marginally reflects a distributional shift” (p. 1364).

However, this claim is somewhat difficult to maintain in light of the findings of Piai et al. (2011; Piai, Roelofs, & Schriefers, 2012), who obtained semantic interference effects in the μ parameter in two different experiments. Taken together, the empirical results would seem to suggest that there is not a simple mapping between semantic interference and distributional parameters, contrary to what Scaltritti et al. (2015) maintain. Rather, semantic interference may be present in the μ or τ parameter depending on the experimental circumstances.

In defence of their claim of a selective mapping, Scaltritti et al. (2015) argued that the findings of Piai et al. (2011; Piai, Roelofs, & Schriefers, 2012) should be taken with caution, because their experiments were not specifically designed to examine how the effect of semantic interference is reflected in the ex-Gaussian parameters. Whereas the aim of Piai et al. (2011) was to examine the effect of task decisions on the presence or absence of semantic interference, Piai, Roelofs, and Schriefers (2012) examined the role of distractor visibility. This may have led to some unusual experimental parameters. For instance, in Piai et al. (2011), the picture–word stimuli were presented for only 250 ms, whereas Piai, Roelofs, and Schriefers (2012) presented the distractor words for only 53 ms. These presentation durations are clearly different from the durations commonly used in the picture–word interference literature. In particular, picture–word stimuli typically remain present throughout (most of) a trial (e.g., W. R. Glaser & Düngelhoff, 1984; W. R. Glaser & Glaser, 1989; Rayner & Springer, 1986; Starreveld & La Heij, 1996; but see Damian & Martin, 1999). Although Piai et al. (2011; Piai, Roelofs, & Schriefers, 2012) obtained semantic interference in the mean RTs, it cannot be excluded that the short stimulus duration in their experiments has contaminated the results from the ex-Gaussian analyses. For example, Piai, Roelofs, and Schriefers (2012) obtained a semantic interference effect in μ when the distractor words were poorly visible and an effect in τ when they were clearly visible. The picture–word interference experiments of Scaltritti et al. were conducted using the common long stimulus presentation duration. Thus, it remains possible that under normal experimental circumstances, semantic interference is present in the τ parameter only, providing support for the claim of Scaltritti et al. that semantic interference is selectively linked to the tail of an RT distribution.

To examine this possibility, we conducted a picture–word interference experiment with a common stimulus duration and assessed the distributional properties of semantic interference by conducting an ex-Gaussian analysis. Moreover, to be able to compare the results with those of colour–word Stroop task performance (i.e., Heathcote et al., 1991; Mewhort et al., 1992; Roelofs, 2012; Spieler et al., 1996, 2000), we also analysed Stroop-like interference and facilitation effects. In addition, to make sure that our findings generalize to other materials and participants, we also conducted an ex-Gaussian analysis on the RT data of another study—namely Piai, Roelofs, and Van der Meij (2012)—for reasons explained later.

## Experimental study

### Method

#### Participants

The experiment was carried out with 16 paid participants, who were students at Radboud University, Nijmegen. All were young adults and native speakers of Dutch.

#### Materials and design

The materials consisted of 32 pictured objects from eight different semantic categories together with their basic-level names in Dutch, listed in the Appendix. The pictures were line drawings, which were scaled to fit into a virtual frame of 10 cm × 10 cm. The distractor words were presented in 36-point lower-case Arial font. Picture and word were presented in white on a black background.

Each target picture was combined with the corresponding word (identity), a word from the same semantic category (semantic), a word from another semantic category (unrelated), or five Xs (neutral). The semantic and unrelated conditions were created by recombining pictures and words (see Appendix). All pictures and words occurred equally often in all conditions. A participant received 32 picture–distractor pairings in each of the four distractor conditions. Each picture–distractor combination was repeated three times in the experiment. The order of presenting the stimuli across trials was random, except that repetitions of pictures and words on successive trials were not permitted.

#### Apparatus and procedure

The experiment was run under the Nijmegen Experiment Setup on a PC. RTs were measured using an electronic voice key. The participants were tested individually. They were seated in front of a computer monitor and a microphone. The distance between participant and screen was approximately 66 cm. Participants were given written instructions telling them that they had to name the picture of picture–word stimuli as quickly as possible while trying to make no mistakes. Before testing, the participants received a booklet with the pictures and their names.

Following the instructions, a block of 32 practice trials was administered, in which all pictures were named once. This was followed by the 384 experimental trials. A trial started by the presentation of a picture–word pair, which remained on the screen for 1500 ms. RTs were measured from stimulus onset. Before the start of the next trial there was a blank interval of 2.5 s.

#### Analyses

A naming response was considered to be invalid when it included a speech error, when a wrong word was produced, or when the voice key was triggered incorrectly. Error trials were discarded from the analyses of the RTs. Mean RTs were submitted to repeated measures analysis of variance (ANOVA) to test for an effect of distractor condition. The analyses were performed both by participants (*F*_1_) and by items (*F*_2_) adopting an alpha level of .05. For the planned comparisons assessing the semantic interference, Stroop-like interference, and Stroop-like facilitation effects, dependent *t* tests were performed by participants (*t*_1_) and by item (*t*_2_) with the alpha level adjusted for three comparisons (i.e., .017). Given that the directions of the effects were predicted, the tests were one-tailed.

The ex-Gaussian parameters were estimated per distractor condition for each participant individually using the quantile maximum likelihood method and the QMPE software of Brown and Heathcote (2003). All estimations converged within 21 iterations. The parameters were submitted to repeated measures ANOVAs. For the planned comparisons to assess the effects in each ex-Gaussian parameter, dependent *t* tests were used with the alpha level adjusted for three comparisons (i.e., .017). Again, the tests were one-tailed.

### Results

#### Analysis of means

Table 1 shows that mean RTs were longer on semantic than on unrelated trials (semantic interference), longer on semantic than on neutral trials (Stroop-like interference), and slightly shorter on identity than on neutral trials (Stroop-like facilitation). Error rates were higher on semantic than on other trials, which did not differ from each other. Most errors were made in the slowest condition, excluding a speed–accuracy trade-off.

**Table 1.** Mean response time, percentage error, and mean ex-Gaussian parameter estimates as a function of distractor condition

Distractor condition	*M*	PE	μ	σ	τ
Semantic	869 (25)	2.8 (0.6)	714 (23)	66 (9)	155 (10)
Unrelated	830 (25)	1.5 (0.4)	696 (20)	51 (6)	130 (11)
Neutral	741 (19)	1.6 (0.3)	619 (18)	45 (4)	123 (10)
Identity	727 (21)	1.5 (0.4)	611 (18)	45 (5)	116 (10)

*Note: M* = mean response time; PE = percentage error; μ, σ, τ*=* mean ex-Gaussian parameter estimates. Mean response times and ex-Gaussian parameter estimates are given in milliseconds. Standard errors of the mean are shown in parentheses.

The statistical analysis of the RTs yielded an effect of distractor condition [*F*_1_(3, 45) = 117.83, *MSE* = 637, *p* < .001, $\eta_{p}^{2}$ = .89; *F*_2_(3, 93) = 99.27, *MSE* = 1546, *p* < .001, $\eta_{p}^{2}$ = .76]. Planned comparisons revealed that responding was slower on semantic than on unrelated trials [*t*_1_(15) = 11.09, *MSE* = 95, *p* < .001, $\eta_{p}^{2}$ = .89; *t*_2_(31) = 4.14, *MSE* = 1382, *p* < .001, $\eta_{p}^{2}$ = .36]. Thus, the standard semantic interference effect was obtained. Moreover, RTs were longer on semantic than on neutral trials [*t*_1_(15) = 12.55, *MSE* = 822, *p* < .001, $\eta_{p}^{2}$ = .91; *t*_2_(31) = 10.01, *MSE* = 2635, *p* < .001, $\eta_{p}^{2}$ = .76], but there was no statistically reliable difference between identity and neutral trials [*t*_1_(15) = 1.50, *MSE* = 691, *p* = .08, $\eta_{p}^{2}$ = .13; *t*_2_(31) = 2.59, *MSE* = 487, *p* < .007, $\eta_{p}^{2}$ = .18]. Thus, Stroop-like interference was obtained, but there was no reliable evidence for Stroop-like facilitation.

#### Distributional analysis

**Figure 1.**
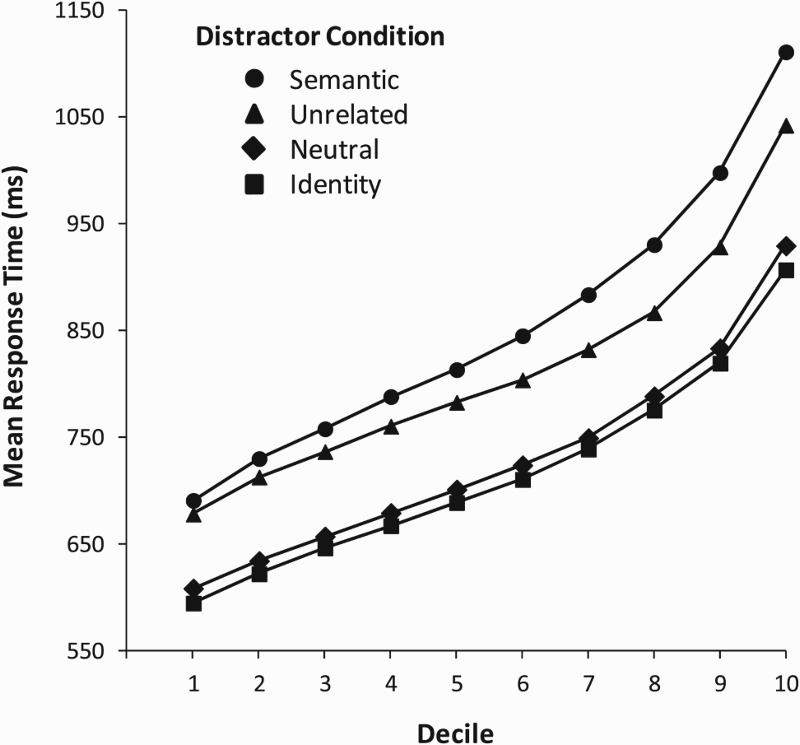
Mean response time as a function of decile per distractor condition in the experiment.

Figure 1 gives the mean RT as a function of decile per distractor condition. The distributions were obtained by rank-ordering (from fastest to slowest) the condition RTs for each participant, dividing the rank-ordered RTs into deciles, and averaging the decile means across participants (e.g., Balota et al., 2008; Ratcliff, 1979). The figure shows that the semantic and unrelated conditions differed throughout the entire RT distribution, although the difference was small for the 10% fastest responses and became larger for the slower responses. Table 1 gives the ex-Gaussian parameter estimates per distractor condition.

**Figure 2.**
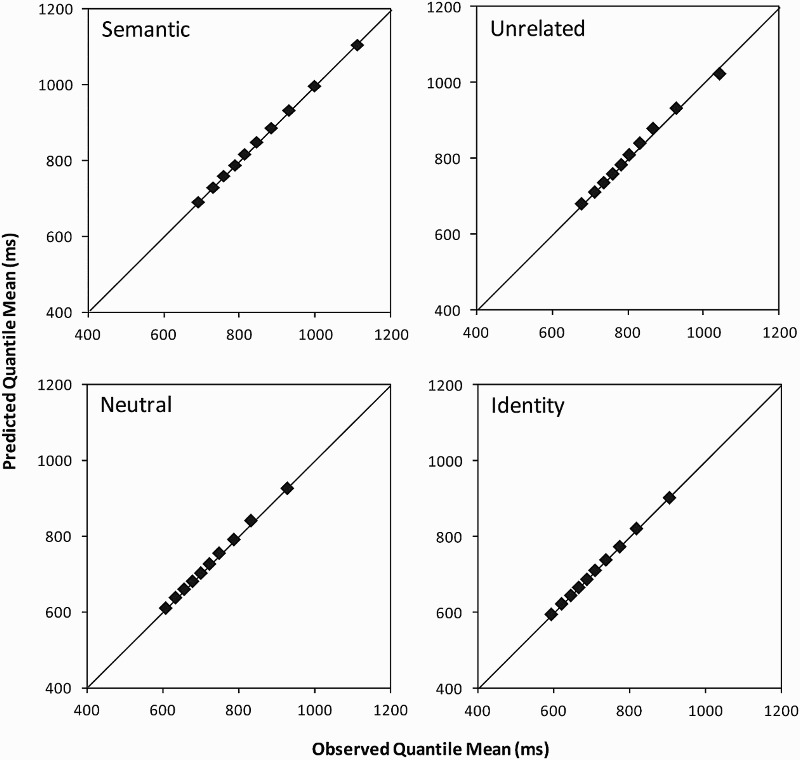
Quantile–quantile (Q–Q) plots for each distractor condition in the experiment. For each decile, the mean response time predicted by the estimated ex-Gaussian parameters is plotted against the empirically observed mean response time.

To graphically evaluate the goodness of fit between the ex-Gaussian and empirical RT distributions, Figure 2 shows quantile–quantile (Q–Q) plots for each condition (cf. Steinhauser & Hübner, 2009). For each decile, the mean RT predicted by the estimated ex-Gaussian parameters is plotted against the empirically observed mean RT (shown in Figure 1). The better the goodness of fit, the closer the points in the Q-Q plots are to the diagonal line. The figure slows that the goodness of fit for the different distractor conditions is good.

Statistical analysis revealed that there was a main effect of distractor condition in μ, *F*(3, 45) = 91.54, *MSE* = 486, *p* < .001, $\eta_{p}^{2}$ = .86. Planned comparisons revealed that μ was larger for semantic than for unrelated trials (semantic interference), *t*(15) = 2.42, *MSE* = 428, *p* < .015, $\eta_{p}^{2}$ = .28. [The semantic interference effect was also present in μ when the ex-Gaussian parameters were estimated using the continuous maximum likelihood method and the QMPE software, *t*(15) = 2.69, *MSE* = 657, *p* < .01, $\eta_{p}^{2}$ = .32.] Moreover, μ was larger for semantic than for neutral trials (Stroop-like interference), *t*(15) = 9.70, *MSE* = 761, *p* < .001, $\eta_{p}^{2}$ = .86, but not reliably smaller for identity than for neutral trials (Stroop-like facilitation) when corrected for multiple comparisons, *t*(15) = 1.95, *MSE* = 170, *p* = .04, $\eta_{p}^{2}$ = .20. Thus, the statistical analysis shows that the semantic and Stroop-like interference effects were reflected by distributional shifting. There was no statistically reliable effect of distractor condition in σ, *F*(3, 45) = 2.79, *MSE* = 530, *p* = .051, $\eta_{p}^{2}$ = .16. Finally, there was a main effect of distractor condition in τ, *F*(3, 45) = 7.18, *MSE* = 637, *p* < .001, $\eta_{p}^{2}$ = .32. Planned comparisons revealed that τ was larger for semantic than for unrelated trials (semantic interference), *t*(15) = 2.80, *MSE* = 610, *p* < .007, $\eta_{p}^{2}$ = .34, larger for semantic than for neutral trials (Stroop-like interference), *t*(15) = 4.36, *MSE* = 436, *p* < .001, $\eta_{p}^{2}$ = .56, but not smaller for identity than for neutral trials (Stroop-like facilitation), *t*(15) < 1, *MSE* = 775, *p* = .52, $\eta_{p}^{2}$ = .03. To summarize, semantic and Stroop-like interference effects were obtained in μ and τ, while Stroop-like facilitation was observed in neither μ nor τ.

Following Scaltritti et al. (2015), we also performed analyses of variance on the quantiles for the semantic interference effect, considering decile and distractor type (semantic vs. unrelated) as within-participants and within-items factors. The interaction between the two factors was significant in the by-participants analysis only [*F*_1_(9, 135) = 12.73, *MSE* = 295, *p* < .001, $\eta_{p}^{2}$ = .46; *F*_2_(9, 279) = 1.42, *MSE* = 908, *p* = .18, $\eta_{p}^{2}$ = .04]. Planned comparisons revealed that the semantic interference effect was already present in the first decile (i.e., 13 ms) [*t*_1_(15) = 3.42, *MSE* = 113, *p* < .002, $\eta_{p}^{2}$ = .44; *t*_2_(31) = 4.68, *MSE* = 448, *p* < .001, $\eta_{p}^{2}$ = .41].

To summarize, we replicated the standard semantic and Stroop-like interference effects in the mean picture naming RTs. Ex-Gaussian analysis showed that these effects were reflected in μ and τ. Quantile analysis revealed that the semantic interference effect was already present in the fastest responses but varied with decile in the by-participants analysis only. Stroop-like facilitation was not reliably present in the mean RTs, μ, and τ. Our results converge with those of Scaltritti et al. (2015) in that the semantic interference effect was larger for the longer than for the shorter RTs. This was evident from the semantic interference effect in τ and the (by-participants) interaction between decile and distractor type, which replicates the findings of Scaltritti et al. However, Scaltritti et al. did not find a semantic interference effect in μ, whereas such an effect was obtained in the present experiment.

### Discussion

Scaltritti et al. (2015) observed semantic interference mainly in the τ parameter and obtained only full-blown semantic interference in the slowest deciles. Based on these findings, they argued that semantic interference is selectively linked to the tail of an RT distribution. Our finding of a semantic interference effect in μ challenges this claim. Whereas previous studies that obtained semantic interference in μ employed somewhat uncommon stimulus presentation durations (Piai et al., 2011; Piai, Roelofs, & Schriefers, 2012), in the present experiment we used a standard stimulus duration. Still, we observed semantic interference in μ. This excludes the possibility that the previous findings of semantic interference in μ are due to the unusual stimulus durations. However, a major methodological difference between our present study (and Piai et al., 2011; Piai, Roelofs, & Schriefers, 2012) and Scaltritti et al.'s is that our distractor words were all names of pictures in the experiment, whereas this was not the case in the study of Scaltritti et al. In their experiments, the distractor words in the semantically related and unrelated conditions were not part of the response set. To examine whether response set membership is the crucial factor causing the difference in results between studies, we performed an ex-Gaussian analysis on the RT data of Piai, Roelofs, and Van der Meij (2012), who had not done this analysis before. Piai, Roelofs, and Van der Meij (2012) conducted a picture–word experiment with semantic, unrelated, and identity conditions, whereby the distractor words in the semantic and unrelated conditions were not part of the response set, exactly as in the study of Scaltritti et al. If semantic interference in μ is due to the response set membership of the distractor words in our study, the ex-Gaussian analysis of the data of Piai, Roelofs, and Van der Meij (2012) should yield semantic interference in τ but not in μ, just as Scaltritti et al. observed.

### Analysis of Piai, Roelofs, and Van der Meij (2012)

For all the experimental details, we refer to Piai, Roelofs, and Van der Meij (2012). Their picture–word interference experiment had semantic, unrelated, and identity conditions. As before, ex-Gaussian parameters were estimated per distractor condition (i.e., semantic, unrelated, identity) for each participant individually using the quantile maximum likelihood method and the QMPE software of Brown and Heathcote (2003). All estimations converged within 21 iterations. The parameters were submitted to repeated measures ANOVAs. For the planned comparisons assessing the semantic interference effect in the ex-Gaussian parameters, dependent *t* tests were used with the alpha level at .05. Given that the direction of the effect was predicted, the tests were one-tailed.

**Figure 3.**
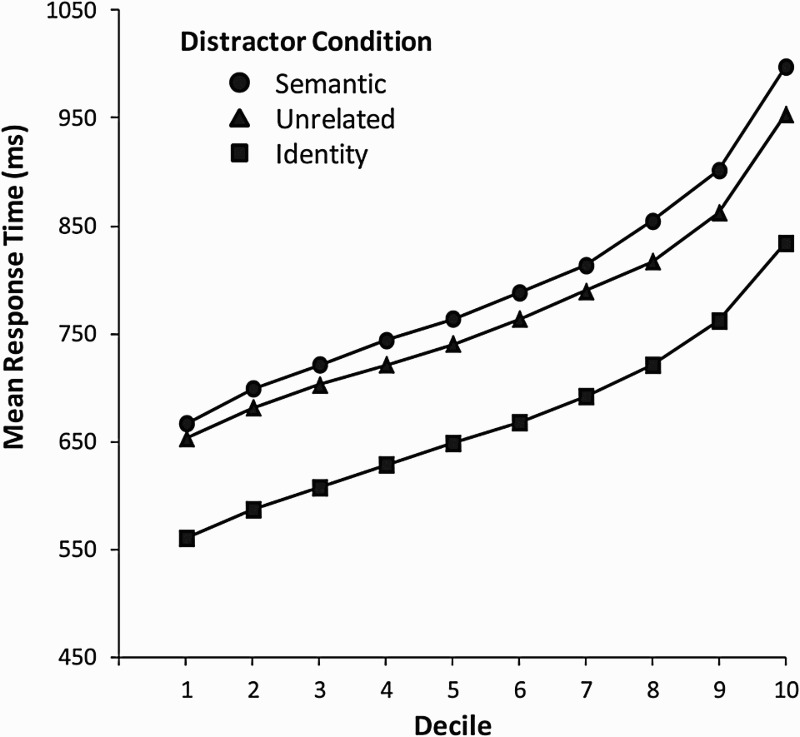
Mean response time as a function of decile per distractor condition in the experiment of Piai, Roelofs, and Van der Meij (2012).

**Figure 4.**
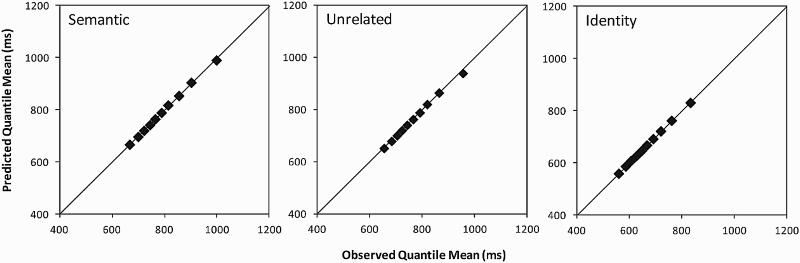
Quantile–quantile (Q–Q) plots for each distractor condition in the experiment of Piai, Roelofs, and Van der Meij (2012). For each decile, the mean response time predicted by the estimated ex-Gaussian parameters is plotted against the empirically observed mean response time.

Figure 3 gives the mean RT as a function of decile per distractor condition. The figure shows that the semantic and unrelated conditions differed throughout the entire RT distribution, although the difference was small for the 10% fastest responses and became larger for the slower responses. Figure 4 shows the Q–Q plots for each distractor condition, revealing that the goodness of fit for the different conditions is good.

The values for μ in the semantic, unrelated, and identity conditions were 683, 669, and 581 ms (with standard errors of 11, 11, and 12 ms), respectively. Statistical analysis revealed that there was a main effect of distractor condition in μ, *F*(2, 38) = 192.68, *MSE* = 319, *p* < .001, $\eta_{p}^{2}$ = .91. A planned comparison revealed that μ was larger for semantic than for unrelated trials, *t*(19) = 3.52, *MSE* = 146, *p* < .001, $\eta_{p}^{2}$ = .39. [The semantic interference effect was also present in μ when the ex-Gaussian parameters were estimated using the continuous maximum likelihood method and the QMPE software, *t*(19) = 4.05, *MSE* = 126, *p* < .001, $\eta_{p}^{2}$ = .46.] The values for σ in the semantic, unrelated, and identity conditions were 51, 46, and 49 ms (all with standard errors of 4 ms), respectively, which did not differ from each other, *F*(2, 38) < 1, *MSE* = 191, *p* = .59, $\eta_{p}^{2}$ = .03. Finally, the values for τ in the semantic, unrelated, and identity conditions were 123, 108, and 98 ms (with standard errors of 9, 9, and 6 ms), respectively, which differed significantly, *F*(2, 38) = 6.09, *MSE* = 524, *p* < .005, $\eta_{p}^{2}$ = .24. A planned comparison revealed that τ was larger for semantic than for unrelated trials, *t*(19) = 2.50, *MSE* = 370, *p* < .01, $\eta_{p}^{2}$ = .25. To summarize, semantic interference was obtained in μ and τ.

We also performed analyses of variance on the quantiles for the semantic interference effect. The interaction between decile and distractor type (semantic vs. unrelated) was significant in the by-participants analysis only [*F*_1_(9, 171) = 4.61, *MSE* = 223, *p* < .001, $\eta_{p}^{2}$ = .20; *F*_2_(9, 351) = 1.25, *MSE* = 662, *p* = .26, $\eta_{p}^{2}$ = .03]. Planned comparisons revealed that the semantic interference effect was already present in the first decile (i.e., 13 ms) [*t*_1_(19) = 4.25 *MSE* = 106, *p* < .001, $\eta_{p}^{2}$ = .49; *t*_2_(39) = 2.90, *MSE* = 717, *p* < .003, $\eta_{p}^{2}$ = .18].

Piai, Roelofs, and Van der Meij (2012) used pictures with high- and low-frequency names. In the analyses above, we collapsed across this frequency factor. However, it remains possible that responses to pictures with high- and low-frequency names are differentially represented across the RT distribution. Arguably, responses to pictures with low-frequency names will be more inclined to be represented within the slower portions of the RT distribution, and vice versa for responses to pictures with high-frequency names. The finding that the semantic interference effect for the first decile was significant not only by participants but also by items already suggests that this effect is not driven by only a subset of the pictures (i.e., those with high-frequency names). To further corroborate this, we conducted the quantile analysis again but now with frequency as a between-items factor. The results showed that there was no interaction of frequency, decile, and distractor condition (i.e., semantic vs. unrelated), *F*_2_(9, 342) < 1, *MSE* = 675, *p* = .99, $\eta_{p}^{2}$ = .006. Moreover, the magnitude of the semantic interference effect in the first decile did not differ between pictures with high- and low-frequency names, *F*_2_(1, 38) = 1.69, *MSE* = 705, *p* = .20, $\eta_{p}^{2}$ = .04. Thus, the semantic interference effect in the fast responses is not confined to a subset of the pictures.

We argued that if the semantic interference in μ in the new study reported above and in the studies of Piai et al. (2011; Piai, Roelofs, & Schriefers, 2012) is due to the response set membership of the distractor words, the ex-Gaussian analysis of the data of Piai, Roelofs, and Van der Meij (2012) should yield semantic interference in τ but not in μ, just as Scaltritti et al. (2015) observed. However, in contrast to this prediction, ex-Gaussian analysis of the RT data of Piai, Roelofs, and Van der Meij (2012) showed that semantic interference was observed in μ and τ. Thus, semantic interference is obtained in μ regardless of the response set membership of the distractor words.

To summarize, the results of Piai, Roelofs, and Van der Meij (2012) converge with those of Scaltritti et al. (2015) in that the semantic interference effect was larger for the longer than for the shorter RTs. This was evident from the semantic interference effect in τ and the (by-participants) interaction between decile and distractor type, which replicates the findings of Scaltritti et al. and the new experiment above. However, Scaltritti et al. did not find a semantic interference effect in μ, whereas such an effect was obtained in the experiment of Piai, Roelofs, and Van der Meij (2012) and in the new experiment.

## General Discussion

In a picture–word interference study, Scaltritti et al. (2015) observed reliable semantic interference in the τ parameter only and a full-blown semantic interference effect only in the slowest deciles. On the basis of these findings, they argued that semantic interference is selectively linked to the tail of an RT distribution. However, Piai et al. (2011; Piai, Roelofs, & Schriefers, 2012) obtained semantic interference effects in μ. Still, the stimulus presentation durations of these studies were somewhat unusual. This may be the reason why Piai, Roelofs, and Schriefers (2012) obtained a semantic interference effect in μ when the distractor words were poorly visible and an effect in τ when they were clearly visible. In the present study, we therefore examined whether semantic interference is linked to μ, τ, or both, using a common presentation duration and visibility. We obtained semantic interference in both μ and τ. The distractor words in the experiment were all part of the response set (as in Piai et al., 2011, Experiment 1; Piai, Roelofs, & Schriefers, 2012, Experiment 2), unlike what was the case in the study of Scaltritti et al. To examine whether the difference in results between studies is due to response set membership, we analysed the RT data of Piai, Roelofs, and Van der Meij (2012), whose semantically related and unrelated distractor words were also not in the response set. The ex-Gaussian analysis of the data of this earlier study showed that semantic interference was obtained in both μ and τ. Thus, semantic interference in μ is obtained regardless of the response set membership of the distractor words.

Taken together, our present findings and those of Piai et al. (2011; Piai, Roelofs, & Schriefers, 2012; Piai, Roelofs, & Van der Meij, 2012) provide evidence that semantic interference is not selectively linked to the τ parameter, contrary to the claim of Scaltritti et al. (2015). Our results challenge any simple one-to-one mapping between semantic interference and distributional parameters.

The ex-Gaussian is an ad hoc distribution that may be used to capture the influence of experimental manipulations on RT distributions (e.g., Luce, 1986). If an experimental manipulation yields an effect in μ, this means that the manipulation shifted the whole RT distribution in one condition relative to another. Moreover, if an experimental manipulation yields an effect in τ, this means that the effect of the manipulation is one of skewing, so that a larger difference occurs in the slowest than in the faster responses. And if an effect is present in both μ and τ, the manipulation led to both distributional shifting and skewing. Based on their finding of a semantic interference effect in τ only and a full-blown semantic interference effect only in the slowest deciles, Scaltritti et al. (2015) argued that semantic interference occurs only in “those trials in which attention is operating less effectively” (p. 1364). In contrast, the data of Piai et al. (2011; Piai, Roelofs, & Schriefers, 2012; Piai, Roelofs, & Van der Meij, 2012) and the present study indicate that semantic interference is not necessarily present in the distribution tail only but may be present throughout the entire RT distribution as well. In the terminology of Scaltritti et al., this would suggest that semantic interference occurs even when attention is operating effectively but there is a limit to what attention can do. This is in line with the account of semantic interference by the WEAVER++ model, where a semantic manipulation shifts the whole latency distribution in one condition relative to another (see Roelofs, 2008). Figures 1 and 3 show that semantic interference was only small in the 10% fastest responses. This suggests that when response planning is really fast, the distractor is not processed extensively enough to yield a large semantic effect. Or alternatively, response planning is really fast exactly because the distractor is not processed extensively enough to yield a large semantic effect. It may be that distractor processing is not extensive (i.e., yields little semantic activation) because selective attention is operating effectively, as suggested by Scaltritti et al. Regardless of the cause of the smallness of the semantic effect in the fastest responses, our present findings and those of Piai et al. (2011; Piai, Roelofs, & Schriefers, 2012; Piai, Roelofs, & Van der Meij, 2012) provide evidence that semantic interference may occur in μ (i.e., may be reflected by distributional shifting).

Previous and present results indicate that semantic interference may also occur in τ (Piai, Roelofs, & Schriefers, 2012; Piai, Roelofs, & Van der Meij, 2012; Scaltritti et al., 2015). This would suggest that a semantic interference effect that is present on all trials may have an increased magnitude on the slowest trials (as observed by Piai, Roelofs, & Van der Meij, 2012), or an effect that is absent on most of the trials may appear on the slowest trials (as observed by Scaltritti et al., 2015). An increased magnitude on the slowest trials may occur when attention is operating less effectively, which may be related to the inhibition ability of the participants (e.g., Shao, Meyer, & Roelofs, 2013; Shao, Roelofs, Martin, & Meyer, 2015). Moreover, attention waxes and wanes during continuous and repetitive task performance (Wundt, 1918). If attention is less focused on some of the trials than it is on most trials, this would yield a long RT and a larger semantic interference effect on those trials. Similarly, if the power that semantically related distractor words have to interfere in an experiment happens to be low, then semantic interference may be absent on most of the trials and may arise only when attention is operating less effectively, which would then yield a long RT and a semantic interference effect on those trials. Scaltritti et al. (2015) used the same semantically related and unrelated words in both their experiments testing for semantic interference, so it cannot be excluded that their words had, for unknown reasons, low power to semantically interfere. This would then yield a semantic interference effect predominantly in τ (their Experiment 1) or only in τ (their Experiment 3). Regardless of the explanation of the findings of Scaltritti et al., the present results make clear that semantic interference is not necessarily linked only to the τ parameter. Rather, our results indicate that semantic interference may consistently be obtained in μ. Thus, there exists no simple one-to-one mapping between semantic interference and one of the ex-Gaussian parameters (i.e., τ), contrary to what Scaltritti et al. suggest.

We argued that picture–word interference effects are related to colour–word Stroop effects (e.g., W. R. Glaser & Glaser, 1989; Roelofs, 2003). In the Stroop task, mean RT is typically longer on incongruent than on neutral trials (Stroop interference) and often shorter on congruent than on neutral trials (Stroop facilitation). Similarly, in picture–word interference, RTs are longer on semantically related than on neutral trials (Stroop-like interference) and sometimes shorter on identity trials than on neutral trials (Stroop-like facilitation). We replicated the Stroop-like interference effect in the present study, but we obtained no Stroop-like facilitation in the mean RTs. Previous colour–word Stroop studies using ex-Gaussian analyses showed that Stroop interference is present in both μ and τ, whereas Stroop facilitation is present in μ but an opposite effect (i.e., congruent slower than neutral) occurs in τ (Heathcote et al., 1991; Mewhort et al., 1992; Roelofs, 2012; Spieler et al., 1996, 2000). Our ex-Gaussian analysis revealed that Stroop-like interference was present in both μ and τ, whereas a Stroop-like facilitation effect was present in neither μ nor τ. Thus, whereas the Stroop interference in the colour–word task and the Stroop-like interference in the picture–word task are present in both μ and τ, the results for Stroop and Stroop-like facilitation differ. Whereas facilitation tends to be small or absent in the mean RTs for both the colour–word and picture–word task, the colour–word task shows opposing effects in μ and τ, whereas such opposing effects are absent in the picture–word task. Scaltritti et al. (2015) observed a large significant Stroop-like facilitation effect (i.e., RTs were smaller in the identity than in the neutral condition by 40 ms in their Experiment 1). Moreover, their ex-Gaussian analysis showed that this facilitation effect was present in both μ and τ. Thus, the Stroop and Stroop-like facilitation effects in the colour–word and picture–word tasks seem to differ in their reflection in the distributional parameters.

This difference in results between tasks may be related to the fact that colour–word Stroop experiments use only a few colours and words with many repetitions, while picture–word interference experiments use typically a few dozen pictures and words with fewer repetitions (e.g., three or four). Some researchers (Aarts, Roelofs, & Van Turennout, 2009; Roelofs, 2012; Steinhauser & Hübner, 2009) have argued that the interference in τ for congruent versus neutral trials in the Stroop task arises because of task set competition. The absence of such interference in τ for the picture–word task observed by Scaltritti et al. (2015) and the present experiment would then suggest that such task set competition is absent in this task, perhaps because of the larger number of stimuli or the lower number of repetitions. This may be examined in future research.

To conclude, a previous picture–word interference study by Scaltritti et al. (2015) linked semantic interference in picture naming to the tail of the underlying RT distribution. In the present study, we replicated the semantic interference in the mean picture naming RTs. Distributional analysis of the RTs and those of a previous study (Piai, Roelofs, & Van der Meij, 2012) revealed that the semantic interference effect was reflected in both μ and τ. These results provide evidence that semantic interference is not selectively linked to the τ parameter, and they warn against any simple one-to-one mapping between semantic interference and distributional parameters.

## ORCID

*Vitória Piai* http://orcid.org/0000-0002-4860-5952

## Appendix

### Materials of the experiment

**Table:**

		Distractor		
Picture		Semantic	Unrelated	Identity
animals	zwaan (swan)	schildpad	rok	zwaan
	schildpad (tortoise)	zwaan	beker	schildpad
	konijn (rabbit)	hert	kerk	konijn
	hert (deer)	konijn	bureau	hert
clothing	trui (sweater)	rok	dolk	trui
	rok (skirt)	trui	zwaan	rok
	hemd (shirt)	jas	oor	hemd
	jas (coat)	hemd	kasteel	jas
transportation	fiets (bike)	trein	kast	fiets
	trein (train)	fiets	arm	trein
	auto (car)	vliegtuig	been	auto
	vliegtuig (plane)	auto	glas	vliegtuig
buildings	molen (windmill)	fabriek	kan	molen
	fabriek (factory)	molen	neus	fabriek
	kasteel (castle)	kerk	jas	kasteel
	kerk (church)	kasteel	konijn	kerk
weapons	dolk (dagger)	zwaard	trui	dolk
	zwaard (sword)	dolk	tafel	zwaard
	kanon (cannon)	pistool	bord	kanon
	pistool (pistol)	kanon	bed	pistool
kitchenware	beker (cup)	kan	schildpad	beker
	kan (jug)	beker	molen	kan
	glas (glass)	bord	vliegtuig	glas
	bord (plate)	glas	kanon	bord
furniture	tafel (table)	kast	zwaard	tafel
	kast (cupboard)	tafel	fiets	kast
	bed (bed)	bureau	pistool	bed
	bureau (desk)	bed	hert	bureau
body parts	arm (arm)	neus	trein	arm
	neus (nose)	arm	fabriek	neus
	been (leg)	oor	auto	been
	oor (ear)	been	hemd	oor
